# Exploring the Characteristics of Atoxigenic *Aspergillus flavus* Isolates and Their Biocontrol Impact on Soil Fungal Communities

**DOI:** 10.3390/jof11070491

**Published:** 2025-06-27

**Authors:** Yanyan Zhang, Wanning Wang, Chenggui Piao, Wenjin Li, Peter J. Cotty, Shihua Shan, Usman Rasheed, Quirico Migheli, Qing Kong

**Affiliations:** 1 School of Food Science and Engineering, Ocean University of China, Qingdao 266404, China; zyany2719@163.com (Y.Z.); wwn990914@163.com (W.W.); servantofthemysteries@gmail.com (C.P.); cottypj@gmail.com (P.J.C.); 2 Tai’an Academy of Agricultural Sciences, Tai’an 271018, China; nkylwj@163.com; 3 Shandong Peanut Research Institute, Qingdao 266100, China; shansh1971@163.com; 4 Institute of Applied Microbiology, College of Agriculture, Guangxi University, Nanning 530005, China; rasheus@outlook.com; 5 Department of Agricultural Sciences, University of Sassari, 07100 Sassari, Italy; qmigheli@uniss.it; 6 Desertiffcation Research Centre (NRD), University of Sassari, 07100 Sassari, Italy

**Keywords:** aflatoxin, atoxigenic *Aspergillus flavus*, biological control, soil fungal community

## Abstract

*Aspergillus flavus* can produce aflatoxins, posing a threat of contamination to peanuts. To mitigate this issue, the use of biocontrol isolates, which do not produce aflatoxins (AF^−^), has been considered to reduce aflatoxin levels. In this study, we evaluated five different AF^−^ isolates belonging to different vegetative compatibility groups, all of which exhibited varying degrees of deletion in aflatoxin biosynthesis gene clusters. One isolate that exhibited poor competitive ability against toxigenic *A. flavus* was eliminated, and the remaining four isolates were formulated as biocontrol agents and applied to a peanut field in Tai’an, Shandong, as a combination. Three months after application, the soil aflatoxin content was reduced from 0.62 ± 0.01 to 0.19 ± 0.03 μg/kg (inhibition rate: 69.35%). Among filamentous fungi in the soil, the proportion of AF^−^ isolates increased from 0% to 4.33%. Using SSR-specific primers, the microbial agents were recovered. We discovered that among the four AF^−^ isolates, CA04 had a lower colonization rate compared to the other three (only 12.00% of the total AF^−^ population), suggesting that the absence of sclerotia may result in poor reversibility and weaker dispersal ability. We utilized Illumina sequencing to investigate the changes in soil fungal ecology. The results showed a reduction in the population density of harmful fungi, such as *Fusarium* spp. (66.18%) and *Plectosphaerella* spp. (79.90%), but an increase in the density of *Nothopassalora personata*. This is the first study on the dispersal distance and soil fungal community structure following the application of AF^−^ agents in peanut fields in China.

## 1. Introduction

Aflatoxin contamination is a significant threat to both agricultural economies and to the health of both humans and animals. This challenging food safety problem affects a variety of crops, including corn [1], cottonseed, and peanuts [2], leading to economic losses both in crop markets and in reduced animal productivity and suitability of animal products for human consumption. China is the main producer of peanuts, which are a primary source of cooking oil, a popular snack, and an important ingredient of many foods and feeds [3], and, as such, peanut aflatoxin content is of particular importance to China [4]. Aflatoxin contamination in peanuts is most frequently attributed to kernel infection by toxigenic *Aspergillus flavus* [5]. Soil serves as a primary source for *A. flavus* inoculum, and severe drought, delayed harvest, and poorly controlled storage conditions contribute significantly to aflatoxin contamination in peanuts [6].

*A. flavus* typically produces sclerotia, mycelial survival structures, under severe environmental conditions [7]. Based on the size of sclerotia and the quantity produced, *A. flavus* can be categorized into two morphotypes: L-type isolates with sclerotia diameters > 400 μm and S-type isolates with sclerotia diameters < 400 μm. Generally, S-type isolates are more toxigenic, and atoxigenic *Aspergillus flavus* typically belongs to the L-morphotype [8]. Apart from aflatoxins, *A. flavus* produces other metabolites, including cyclopiazonic acid (CPA), a specific inhibitor of calcium-dependent ATPase in the sarcoplasmic reticulum, resulting in increased muscle contraction in animals and an effective inducer of plant cell death [9,10].

Current methods to control aflatoxin contamination involve integrated pest management (IPM), physical control, chemical control, and biological control. Although efforts have been made to develop crops with resistance to aflatoxin contamination through breeding and genetic engineering, commercially resistant varieties are yet to be widely available. Similarly, chemical control methods have been ineffective in many cases, but certain organic reagents, such as organic acids and essential oils, have shown promise in reducing aflatoxin production. For example, studies have shown that organic acids suppress aflatoxin production by lowering the expression of aflatoxin biosynthesis-related genes in *A. flavus* [11]. Additionally, natural essential oils and phenolic acids have been found to inhibit the growth of *Aspergillus parasiticus* and aflatoxin production [12]. However, several atoxigenic isolate-based biocontrol products proved highly effective and are in commercial use [13]. Despite their efficacy as biocontrol agents, no such products are approved for use in China. Biological control strategies may be beneficial as they are based on natural components of agroecosystems, minimizing the use of synthetic chemicals and reducing potential harm to the environment [14]. These strategies utilize various microorganisms, including *Bacillus* [15], *Lactobacillus* [16], and AF^−^ isolates [17] to prevent the production of aflatoxins. Using AF^−^ isolates as biocontrol agents results in a modification of the *A. flavus* community structure, with the incidence of aflatoxin producers greatly diminished. This represents a promising approach to reduce aflatoxin contamination in crops [18].

Europe, Africa, and North America [19,20,21] have been at the forefront of implementing AF^−^ isolates as active ingredients in biological control agents (BCAs). Deployment of AF^−^ isolates requires selection and characterization of AF^−^ to be used as BCA active ingredients. This includes isolation and molecular- and field-based analyses to identify promising AF^−^ isolates with competitive exclusion capabilities [22]. Research has delved into the ecological dynamics between toxigenic and AF^−^ isolates, revealing colonization patterns, nutrient competition, and microbial interactions in various crops [23]. AF^−^ isolates not only reduced aflatoxin contamination through multiple mechanisms, such as competition for space and nutrients, but also altered the composition of the *A. flavus* community, allowing atoxigenic *A. flavus* to predominate during crop infections [24]. Field trials and related studies have demonstrated the effectiveness of non-toxic isolates in reducing aflatoxin contamination in crops like maize, peanuts, and pistachios [25,26,27].

The atoxigenic phenotype of many AF^−^ isolates is stable for millennia, with atoxigenicity being retained long enough for multiple lesions in the aflatoxin biosynthesis gene cluster, each sufficient to independently cause atoxigenicity to occur [28]. Atoxigenicity is also stable after application to commercial fields for aflatoxin management. However, novel AF^−^ isolates being developed as active ingredients of BCAs need to be evaluated for stability either through examination of the genetic group in the target environment or through monitoring after applications. Genetic groups in *A. flavus* are delimited by variation at vegetative compatibility loci, with genotypes identical at these loci belonging to the same vegetative compatibility group (VCG). *A. flavus* has many VCGs, which can be delimited through complementation of nitrate non-utilizing (nit^−^) mutants [29]. Vegetative compatibility analyses (VCA) can be used to monitor biocontrol active ingredients after application. Simple sequence repeats (SSRs), also known as microsatellites, have been used to assess the genetic diversity of *A. flavus*, monitor gene flow, and assess linkage equilibria within populations [30]. SSR technology also allows for specific identification of atoxigenic *A. flavus* active ingredients.

Metagenomic analysis of soil fungal communities can assess soil health and predict potential disease outbreaks, thereby optimizing soil management to reduce disease and increase crop yields. Ajilogba et al. [31] conducted metagenomic analysis of soil in Bambara groundnut cultivation and found that the presence of beneficial bacteria in the root zone contributed to increased peanut yields. Potential effects of applying AF^−^ isolates on the soil microflora have not been explored in detail. BCA applications may promote the growth of beneficial microorganisms or inhibit harmful ones. Such questions require investigation through metagenomic analyses. This study systematically studied the properties of AF^−^ isolates native to China and prepared a fungal agent for peanut field application. The toxin content, isolate diffusion distance, and the impact following the application of the BCA on the population of other microorganisms were explored after the fungal agent was applied.

## 2. Materials and Methods

### 2.1. A. flavus Screening and Culture Conditions

The fungal isolates from soil and maize samples from different regions in eastern China were isolated using the dilution plate technique on Modified Rose Bengal Agar (MRBA) [32]. *A. flavus* NRRL3357, atoxigenic *A. flavus* PA04, and atoxigenic *A. flavus* PA10 are isolates that were previously stored in our laboratory. The *A. flavus* isolates used in the experiment were cultured at 30 °C, and *A. flavus* NRRL3357 was used as the standard producer of aflatoxin.

### 2.2. Morphological Characteristics of Atoxigenic A. flavus

The screened atoxigenic *A. flavus* isolates were inoculated onto potato dextrose agar (PDA) and yeast extract sucrose (YES) media, and incubated at 30 °C for 7 d. Subsequently, the atoxigenic *A. flavus* isolates were inoculated onto Wickerham (WKM) medium [33] and cultured at 30 °C for 14 d to observe the formation of their sclerotia.

### 2.3. Production of Aflatoxin and Cyclopiazonic Acid by Atoxigenic A. flavus

High-performance liquid chromatography (HPLC) was employed to detect aflatoxin production from the screened atoxigenic *A. flavus* isolates [34]. The atoxigenic *A. flavus* isolates were inoculated on YES medium (20 g yeast extract, 150 g sucrose, 5 g MgSO_4_·7H_2_O, 1 mL A&M trace element solution, dissolved in 1 L H_2_O, adjusted to pH 6.5, 2% agar) and cultured at 30 °C for 7 d. Subsequently, 1 g of mycelia and medium mixture was weighed, and aflatoxin was extracted using 5 mL of methanol. The methanol mixture was vortexed and centrifuged to obtain the supernatant. After evaporation to dryness in a high-temperature oven, the dried material was resuspended in 1 mL of methanol and filtered through an aflatoxin immunoaffinity column in preparation for HPLC analysis. The mobile phase was composed of methanol, acetonitrile, and water in a ratio of 20:20:60 (*v*/*v*/*v*). The separation was carried out using a SinoChrom ODS-BP column (5 μm, 4.6 × 200 mm) with a flow rate of 0.8 mL/min. Aflatoxins were detected by fluorescence detection with an excitation wavelength of 360 nm and an emission wavelength of 450 nm.

CPA was detected by thin-layer chromatography (TLC) in atoxigenic *A. flavus*. Following the method of Chang et al. [35], atoxigenic *A. flavus* isolates were inoculated on WKM medium (2 g yeast extract, 3 g peptone, 5 g corn starch powder, 2 g glucose, 30 g sucrose, 2 g NaNO_3_, 1 g K_2_HPO_4_·3H_2_O, 0.5 g MgSO_4_·7H_2_O, 0.2 g KCl, 0.1 g FeSO_4_·7H_2_O, dissolved in 1 L H_2_O, adjusted to pH 5.5, 2% agar) and incubated at 30 °C in the dark for 7 d. After cultivation, 1 g of an agar block and mycelia mixture was weighed, and one milliliter of chloroform was added to extract CPA from it. Then, the liquid mixture was transferred to clean microcentrifuge tubes and centrifuged at maximum speed for 2 min to obtain the supernatant. A 200 μL aliquot of the sample was applied to a Si250 silica gel plate (BAKER) for thin-layer chromatography (TLC). The developing solvent for CPA comprises ethyl acetate, methanol, and ammonia water in a volume ratio of 85:15:10 (*v*/*v*/*v*). Following development, the plate was treated with a spray reagent to induce color development. The formulation of the spray reagent consists of 1 g of 4-dimethylaminobenzaldehyde completely dissolved in 75 mL of anhydrous ethanol and 25 mL of concentrated hydrochloric acid.

### 2.4. Vegetative Compatibility Group Analyses

Vegetative compatibility group analysis was conducted on these isolates using the previously described method [36]. The SEL medium (30 g sucrose, 3 g NaNO_3_, 0.5 g KH_2_PO_4_, 0.5 g K_2_HPO_4_, 0.5 g MgSO_4_·7H_2_O, 0.5 g KCl, 25 g KClO_3_, 10 mL Bengal red stock solution, dissolved in 1 L water, adjusted to pH 7.0, 2% agar) was used to screen nitrate-non-utilizing (nit^−^) mutants. A 3 mm well was drilled in the SEL medium, into which 10 μL of a spore suspension (1 × 10^6^ CFU/mL) was inoculated. The culture was incubated at 31 °C for 30 d, with observation starting on the 7th day to select nit^−^ mutants. The screened mutants were inoculated on Czapek’s medium (30 g sucrose, 3 g NaNO_3_, 0.5 g KH_2_PO_4_, 0.5 g K_2_HPO_4_, 0.5 g MgSO_4_·7H_2_O, 0.5 g KCl, 1.0 mL A&M micronutrients, dissolved in 1 L water and adjusted to pH 6.0, with 2% agar), nitrite medium (50 g sucrose, 1 g KH_2_PO_4_, 0.5 g MgSO_4_·7H_2_O, 0.69 g NaNO_2_, dissolved in 1 L water, adjusted to pH 5.5, 2% agar), and hypoxanthine medium (50 g sucrose, 1 g KH_2_PO_4_, 0.5 g MgSO_4_·7H_2_O, 200 mg hypoxanthine, dissolved in 1 L water, adjusted to pH 5.5, 2% agar) for phenotypic analysis, and cultured at 30 °C dark conditions for 3 d to identify niaD^−^, cnx^−^, and nirA^−^ mutants. The niaD^−^ mutant was paired with the cnx^−^ or nirA^−^ mutant, and the spore suspension was inoculated into the 3 mm well in starch agar medium (3 g NaNO_3_, 0.5 g KH_2_PO_4_, 0.5 g K_2_HPO_4_, 0.5 g MgSO_4_·7H_2_O, 0.5 g KCl, 10 g soluble starch, 1 mL A&M micronutrient, 36 g glucose, dissolved in 1 L water, adjusted to pH 6.0, 2% agar), and incubated in the dark at 31 °C for 7 d with a triangular pattern interval of 1 cm. Observations were made to determine whether hyphal fusion occurred in the test pairs, with cross-testing continuing until each isolate was assigned to a VCG.

### 2.5. Cluster Amplification Pattern and Microsatellite Loci Analysis of Atoxigenic A. flavus

DNA was extracted from cultures of atoxigenic *A. flavus* hyphae using the OMEGA Bio-tek Fungal DNA Kit (OMEGA Bio-tek, Norcross, GA, USA). Genomic DNA from NRRL3357, used as a control, along with atoxigenic *A. flavus* isolates and two previously screened atoxigenic isolates, PA04 and PA10, from our laboratory, served as templates for the analysis. Following previously published protocols, polymerase chain reaction (PCR), fragment analysis, and analysis of 17 microsatellite loci were then performed on the fungal isolates [37]. The reaction system of multiplex PCR was composed of 0.08 µmol/L of each primer, AccuStart II PCR SuperMix (Quanta Biosciences, Gaithersburg, MD, USA), and 5 ng of genomic DNA, and sterile water was added for a total volume of 10 µL. The PCR reaction conditions were 94 °C for 1 min, 94 °C for 30 s, 57 °C for 90 s, 72 °C for 30 s, and 60 °C for 30 min, for 30 cycles. Each sample was independently amplified and genotyped at least three times to ensure result reproducibility.

### 2.6. Competitive Analysis Between Atoxigenic A. flavus and A. flavus

#### 2.6.1. Competitive Analysis on Peanuts

Atoxigenic *A. flavus* and toxigenic *A. flavus* isolates were cultivated on PDA medium at 30 °C for 7 d. The spores were washed to create a spore suspension. The spore suspension of atoxigenic *A. flavus* was adjusted to three concentrations: 1 × 10^4^, 1 × 10^5^, and 1 × 10^6^ spores/mL, and the concentrations of NRRL3357 and *A. flavus* isolates were adjusted to 1 × 10^5^ spores/mL. The two types of spore suspensions were mixed in the ratios of 1:10, 1:1, and 10:1 (atoxigenic *A. flavus*: toxigenic *A. flavus*). Peanut kernels were sterilized using 75% ethanol and subsequently washed twice with sterile water. One milliliter of the mixed spore suspension was inoculated into 10 g of peanut kernels in a 250 mL conical flask and cultured for 7 d at 30 °C and 90% humidity.

A precisely weighed 5 g sample was combined with 5 mL of methanol and homogenized at high speed for 20 min using a homogenizer. The mixture was then placed on a shaker set at 30 °C and 200 rpm for 2 h to ensure thorough extraction of aflatoxins with methanol. Afterward, the mixture was filtered through quantitative filter paper to obtain the filtrate. A portion of the filtrate was transferred onto a TLC silica gel plate and developed in the developing solvent. The developing solvent composition for aflatoxins consisted of toluene, ethyl acetate, and acetic acid in a volumetric ratio of 65:35:10 (*v*/*v*/*v*). Finally, the plate was examined under ultraviolet light at 365 nm.

Then, the aflatoxin B_1_ in the samples was quantified using the AFB_1_ ELISA kit (Jiangsu Wise Technology Co., Ltd., Zhenjiang China). The inhibition of aflatoxin biosynthesis was expressed as the inhibition ratio (A) and calculated using Equation (1).

A = (1 − C_1_/C_2_ ) × 100%, (1)

C_1_ is the concentration of AFB_1_ in the sample, and C_2_ is the concentration of AFB_1_ in the control.

#### 2.6.2. Competitive Analysis on PDA Medium

The mixed spore suspension of atoxigenic *A. flavus* and *A. flavus* prepared in Section 2.6.1 (at a ratio of 1:10, 1:1, and 10:1) was evenly spread on PDA medium and incubated at 30 °C for 7 d. One gram of mycelia and medium mixture was weighed, and aflatoxins were extracted using 1 mL of methanol. Following shaking and incubation, the mixture was centrifuged to obtain the supernatant. After evaporating the supernatant to dryness, the remaining dried material was resuspended in chloroform. The resuspended solution was spotted onto a TLC silica gel plate, developed using an aflatoxin developing solvent, and observed under ultraviolet light at 365 nm. Quantitative analysis was performed on AFB_1_ using the above method.

### 2.7. Field Trials

The screened atoxigenic *A. flavus* isolates were incubated on PDA medium at 30 °C for 7 d. Spores were then washed from the medium to prepare a spore suspension of 1 × 10^6^ spores/mL. Corn germ was sterilized at 121 °C for 20 min and subsequently dried overnight at 50 °C to reduce the moisture content to below 8%. A mixture of 100 mL spore suspension and 50 g of corn germ was incubated at 37 °C with 90% humidity for 48 h. The final product was sealed in a polyethylene bag. Four experimental plots were established in Maoguan Village, Laohuguan Village, Daiyue District, Tai’an City, Shandong Province, located at latitude 35.999101° and longitude 117.016344°. Each plot covered an area of 20 m^2^, as displayed in Figure S1. The fungal agent was applied to the soil surface at a dose of 10 kg/ha. Following application, soil samples were collected biweekly from various depths in the trapping plot, including 100 g of surface soil and 100 g of soil at a depth of 8–10 cm, with control samples taken from soil located 200 m away. According to Senghor et al. [38], the application of the fungal agent in this experiment took place on 23 June 2023 (after peanut seed germination) and 5 July 2023 (2–3 weeks before flowering). The peanut variety used in the experiment was HuaYu 39, provided by the Tai’an Academy of Agricultural Sciences. Sample collection times were T1: 20 July 2023, T2: 4 August 2023, and T3: 3 September 2023.

### 2.8. The Processing of Soil Samples

A five-gram soil sample was weighed after the application of the microbial agent, followed by the addition of 95 mL of sterile water. The mixture was shaken at 30 °C and 220 rpm for 30 min and then allowed to stand for 2 h to obtain the supernatant. Subsequently, 30 μL of the supernatant was coated evenly on CU medium and cultured at 30 °C to screen for *A. flavus* colonies. The screened *A. flavus* isolates were inoculated on YES medium and cultured at 30 °C for 7 d to identify toxin production according to the method described in Section 2.3, aiming to screen for AF^−^ isolates. Following the protocol outlined in Section 2.5, DNA extraction and specific amplification were conducted on these AF^−^ isolates, and the size of the amplified fragments was compared with previous SSR results using a nucleic acid protein analyzer to assess whether the applied AF^−^ isolates were successfully recovered. The content of AFB_1_ in harvested peanuts and field soil was determined using the AFB1 ELISA kit (Jiangsu Huisi Technology Co., Ltd., Zhenjiang China). The samples were processed, and the AFB_1_ content was calculated according to the manufacturer’s instructions. For soil samples, microbial DNA from the soil was extracted using the OMEGA E.Z.N.A^TM^ Mag-Bind Soil DNA Kit (OMEGA Bio-tek, Norcross, GA, USA) and DNA concentration was determined using a NANO instrument (LifeReal, Hangzhou, China).

### 2.9. 97% OTU Clustering Biological Classification

After sequencing, the two short Illumina readings were assembled by PEAR software (version 0.9.8). The effective tags were clustered into operational taxonomic units (OTUs) of ≥97% similarity using Usearch software (version 11.0.667). Chimeric sequences and singleton OTUs (with only one read) were removed, after which the remaining sequences were sorted into each sample based on the OTUs. The tag sequence with the highest abundance was selected as a representative sequence within each cluster. The raw reads were deposited into the NCBI Sequence Read Archive (SRA) database (Accession Number: PRJNA1057715).

### 2.10. Data Analysis

All experiments were repeated three times to avoid errors. Powermarker version 3.25 software was used to draw clustering trees, and GraphPad Prism 9. 5. 1 software was used for data analysis. All data were subjected to a one-way ANOVA test in IBM SPSS Statistics 25 software, and *p* < 0.05 was considered statistically significant.

## 3. Results

### 3.1. Sample Fungus Composition and Characteristics of Atoxigenic Isolates

**Figure 1:**
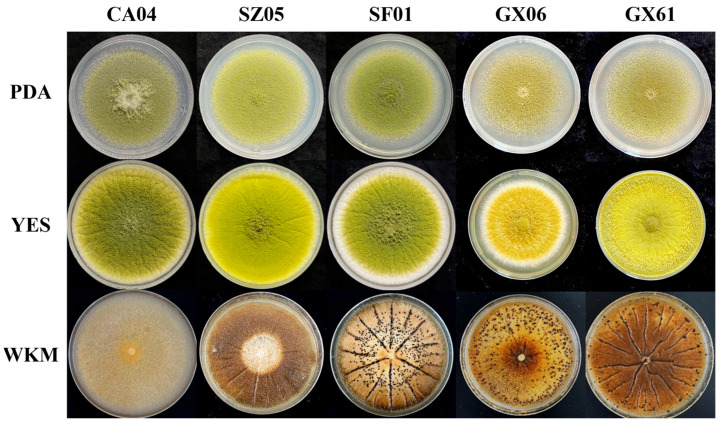
Colonies of atoxigenic *A. flavus* isolates selected from various samples on different media (cultured on YES and PDA media for 7 d, and on WKM medium for 14 d).

In this experiment, 18 isolates of *A. flavus* were isolated from corn samples in Qingdao and soil samples from Fujian and Guangdong (Table 1). All *A. flavus* isolates exhibited the formation of green spores and fluffy hyphae when cultured on PDA medium, as well as dark green or yellow-green spores accompanied by white colony margins on YES medium (Figure 1). There was no significant morphological distinction observed between atoxigenic and toxigenic *A. flavus*. Consequently, based on their capacity for toxin production, five isolates were classified as atoxigenic *A. flavus*, while a single isolate was identified as toxigenic *A. flavus* with a high level of aflatoxin production. Notably, the high aflatoxin-producing *A. flavus* isolate belonged to the S-type isolate group, while the rest of the isolates were classified as L-type isolates. Surprisingly, in the case of atoxigenic *A. flavus*, isolates CA04 and SZ05 were found not to produce sclerotia on either YES and WKM culture media. Furthermore, the VCG analysis revealed that toxigenic and atoxigenic *A. flavus* do not belong to the same VCG. Among the atoxigenic isolates, only GX06 and GX61 demonstrated hyphal fusion and were thus classified within the same VCG.

### 3.2. Detection of Aflatoxins and CPA Production Capability in A. flavus

**Figure 2:**
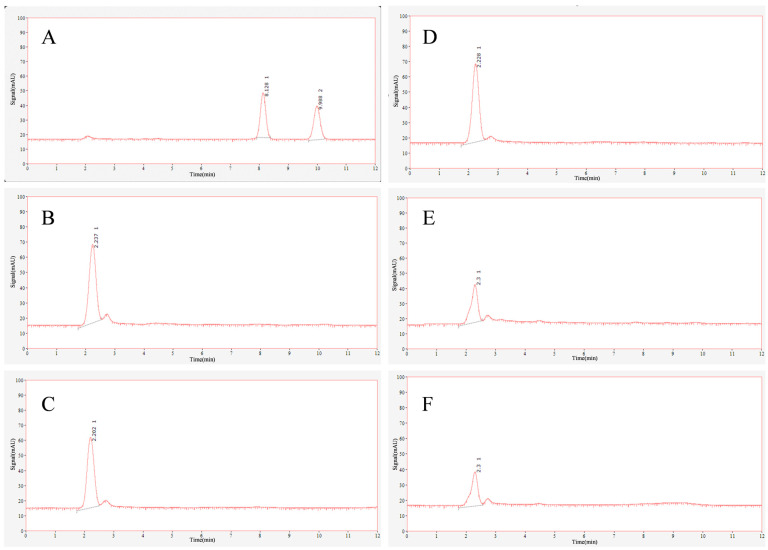
HPLC analysis of isolates CA04 (**B**), SZ05 (**C**), SF01 (**D**), GX06 (**E**), and GX61 (**F**) with AFB_1_ and AFB_2_ standards (**A**) (50 μg/L) as controls.

**Figure 3:**
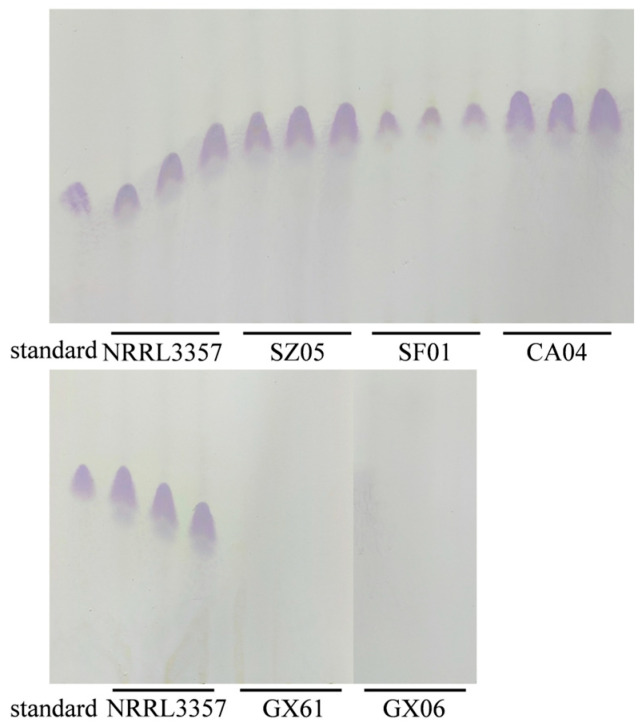
CPA production capability in atoxigenic *A. flavus*.

The aflatoxin production capability of 18 *A. flavus* isolates selected from samples collected from different locations was assessed on YES medium. Notably, five isolates (Figure 2) failed to produce detectable levels of aflatoxins (LOD = 2 μg/kg total aflatoxins). The CPA production was detected on WKM medium. As depicted in Figure 3, it was observed that isolates GX06 and GX61 were incapable of producing CPA, while SF01 showed a limited capacity for CPA production. In contrast, isolates CA04 and SZ05 demonstrated CPA production levels akin to those of NRRL3357.

### 3.3. Cluster Amplification Pattern Analysis and Microsatellite Analysis

**Figure 4:**
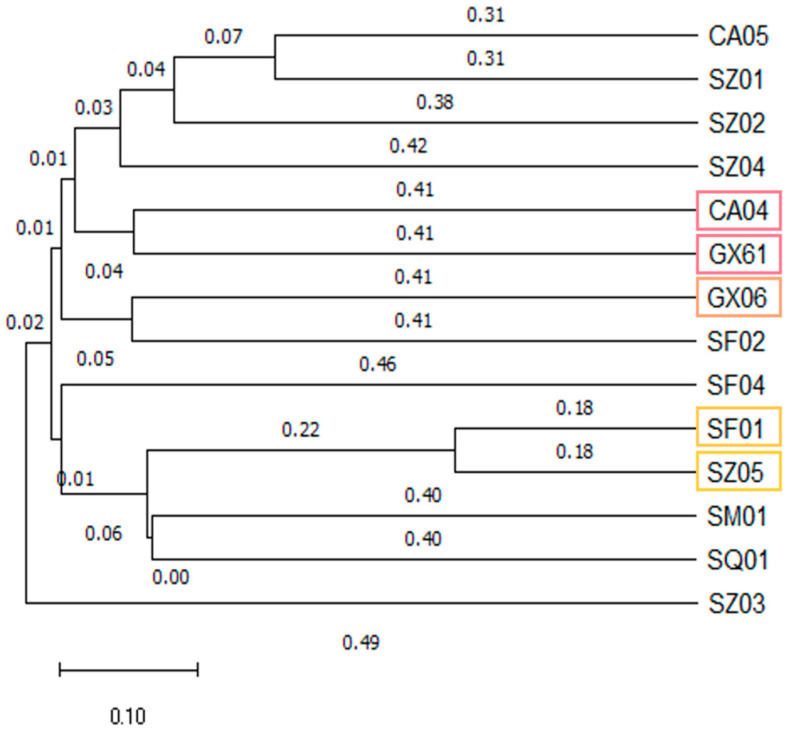
The genetic relatedness among the atoxigenic *A. flavus* isolates.

As indicated in Figure S2, all atoxigenic *A. flavus* isolates exhibited varying degrees of deletions within the aflatoxin biosynthesis gene clusters. To enhance detection efficiency and reduce costs, the construction of a DNA fingerprint map was employed to differentiate between diverse ranges of germplasm resources using a minimal number of primers. As shown in Table S1, when pairwise combinations of SSR primers were utilized, it was possible to identify five characteristic primers for AF^−^ isolates (primers AF43, AF22, AF42, AF11, and AF55). These characteristic primers facilitated the identification of the applied isolates during subsequent field experiments. If the results of the characteristic primer identifications matched, the isolates could be reliably confirmed. In addition, Figure 4 revealed the genetic relatedness among the atoxigenic *A. flavus* isolates. CA04 and GX61, as well as SF01 and SZ05, were closely related, while GX06 exhibited a closer genetic relationship with CA04 and GX61. In contrast, SZ03 displayed distant genetic relatedness when compared to the other *A. flavus* isolates.

### 3.4. Atoxigenic A. flavus Significantly Inhibits the Production of Aflatoxins

**Figure 5:**
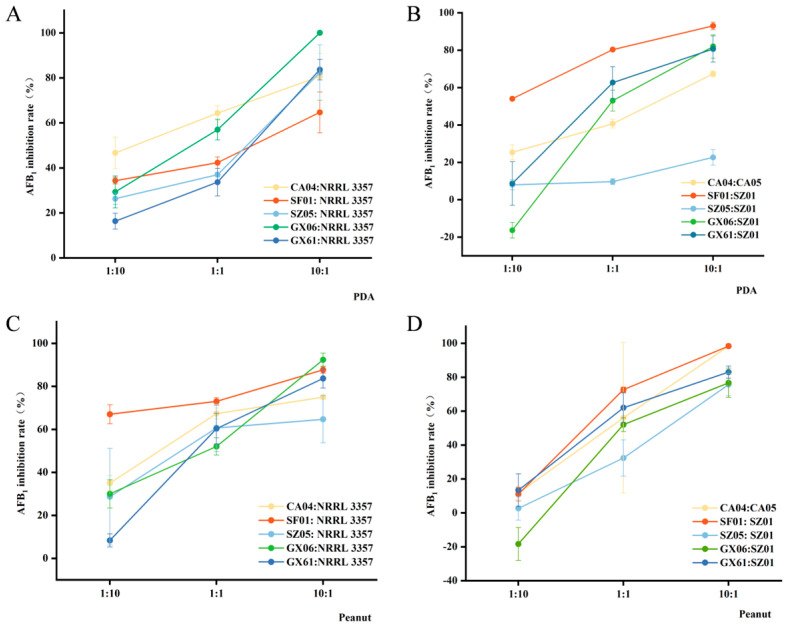
Competition inhibition rates of atoxigenic *A. flavus* inoculated with toxigenic isolates at different concentrations. The inhibition rates of AFB_1_ by co-cultivation of atoxigenic *A. flavus* with NRRL 3357 on PDA medium for 10 d (**A**). The inhibition rates of AFB_1_ by co-cultivation of atoxigenic *A. flavus* with toxigenic isolates from the same screening location on PDA medium for 10 d (**B**). The inhibition rates of AFB_1_ by co-cultivation of atoxigenic *A. flavus* with NRRL 3357 on peanut seeds for 7 d (**C**). The inhibition rates of AFB_1_ by co-cultivation of atoxigenic *A. flavus* with toxigenic isolates from the same screening location on peanut seeds for 7 d (**D**).

Figure 5 and Figure S3 illustrate the inhibition rates of AF^−^ isolates on the wild-type *A. flavus* NRRL 3357 and AF^+^ isolates in terms of AFB_1_ production. It was evident from Figure 5 that, under a 10:1 inoculation ratio, atoxigenic *A. flavus* SF01 exhibited a substantial inhibition of aflatoxin production by the toxigenic *A. flavus* isolates, both on PDA medium and peanut kernels. In contrast, SZ05 demonstrated a lower inhibition rate, which led to the decision not to employ SZ05 in the subsequent field experiments. Overall, when the inoculation ratio was set at 10:1, the average of AF^−^ isolates achieved an inhibition rate of 84.41% of AFB_1_ production by toxigenic *A. flavus* isolates (Table S2).

### 3.5. Field Experiment

**Figure 6:**
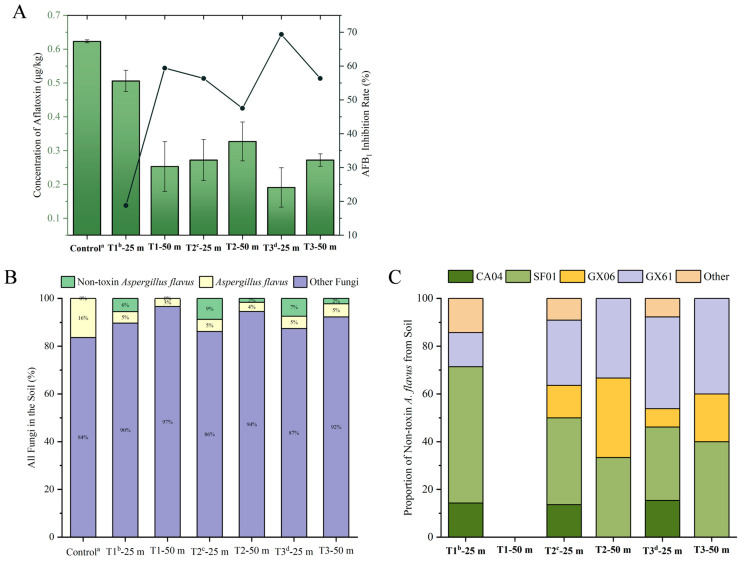
Fungal composition isolated from the soil. The levels of aflatoxins in the soil: The line graph illustrates the AFB_1_ inhibition rate (%), and the bar graph represents the concentration of aflatoxin (μg/kg) (**A**). The proportions of fungi, *A. flavus*, and atoxigenic *A. flavus* (**B**). The proportions of CA04, SF01, GX61, GX06, and wild-type atoxigenic *A. flavus* within the non-toxigenic group (**C**). The a represents soil sampling taken on 23 June 2023, prior to the application of BCAs; b represents the sample taken after the application of the microbial agent on 20 July 2023 (T1); c represents the sampling on 4 August 2023 (T2); d represents the sampling on 3 September 2023 (T3).

Following the application of BCAs in Shandong’s sandy loam soil, the average population of *Aspergillus* spp. in the soil was 4521.77 cfu/g. Due to the overall dry climate, frequent clear skies, and lower precipitation levels in Tai’an, Shandong, during this experiment, the aflatoxin content in the soil was relatively low. The highest recorded level in the untreated group was 0.62 μg/kg. After the application of the BCAs, the aflatoxin content in the soil decreased, with inhibition rates stabilizing at around 50% one month after treatment (Figure 6A).

As seen in Figure 6B, after the application of AF^−^ isolates, the proportion of toxigenic *A. flavus* decreased compared to the 16% observed in the control group. Compared to the soil before biological control agent application, there was an increase in the number of AF^−^ isolates, which reshaped the soil *A. flavus* population structure. These isolates dispersed to soil located at distances of 25 m (two weeks later) and 50 m (one month later) from the point of application, likely aided by wind, precipitation, insects, and animal activities. At 25 m, the proportion of AF^−^ isolates ranged from 6% to 9%, surpassing the proportion of aflatoxin-producing *A. flavus*. This suggests that the AF^−^ isolates in the microbial agents had gained a competitive advantage in their ecological niche within two weeks. At 50 m, AF^−^ isolates were detected one month after application, making up 2% of the population, but this proportion did not exceed that of aflatoxin-producing *A. flavus* at the same distance. The DNA of AF^−^ isolates collected from field trials was extracted and purified, and amplified using SSR primers AF43, AF22, AF42, AF11, and AF55. The amplification products were analyzed using a nucleic acid protein analyzer to determine their fragment length and peak number, and compared with previous experimental results (Table S1). The final results showed that SF01 and GX61 accounted for a large proportion (Figure 6C) of AF^−^ isolates, accounting for 41.60% and 29.98%, respectively. GX06 and CA04, which do not produce sclerotia, had poor effects. The reason may be that GX06 is not adapted to the environment, and CA04 has poor resistance due to its inability to produce sclerotia. If other AF^−^ agents are developed in the future, it is recommended to use isolates that can produce sclerotia, as the production of sclerotia is more conducive to their colonization in the environment.

### 3.6. Analysis of Soil Fungal Composition

Based on the observed OTUs, the fungal community rarefaction curve has reached saturation, indicating that the sequencing depth in this study is sufficient to reflect the microbial diversity in all soil samples (Figure S4A). After standardization, a total of 1457 fungal OTUs were clustered based on a sequence similarity cutoff of 97%. There was no significant change in the number of fungal OTUs detected after the application of non-aflatoxin-producing *A. flavus*. Among the four Trap groups of soil samples, 28 fungal OTUs were shared (Figure S4B).

**Figure 7:**
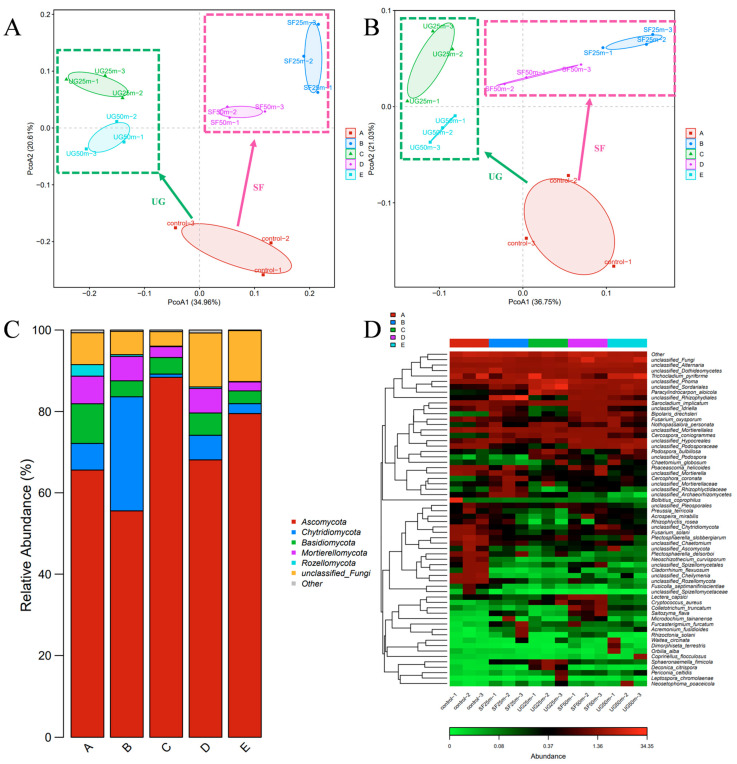
Principal coordinate analysis (PCoA) plots of Bray–Curtis (**A**) and weighted Unifrac (**B**) OTUs based on samples from different distances and depths on the day of peanut harvest, after the application of the microbial agent (SF: soil surface; UG: underground). The figure shows changes in soil fungal distribution before and after the application of biocontrol agents. At the phylum level (**C**) and species level (**D**). The color code represents relative abundance, ranging from green (low abundance) to red (high abundance). A, B, C, D, and E represent the blank control group, the soil surface at 25 m from the microbial agent application site, soil at 10 cm depth 25 m from the application site, the soil surface at 50 m from the application site, and soil at 10 cm depth 50 m from the application site, respectively.

As seen in Figure 7A,B, analysis using the Bray–Curtis and Weighted Unifrac methods revealed differences in fungal population distribution between the control group and the surface and underground soil sampling groups. Metagenomic analysis was employed to monitor potential pathogenic fungi in the soil, enabling the early prediction of crop disease outbreaks. This aids in taking preventive measures to reduce crop losses. In Figure 7C, the most distributed phylum in the soil is *Ascomycota* (70.21%). Compared with BCDE, the number of *Chytridiomycota* (reduced by 90.46%) and *Basidiomycota* (reduced by 23.98%) in the surface layer of soil is significantly greater than that at 10 cm. The application of microbial agents significantly reduced the content of *Basidiomycota*. In Figure 7D, it can be observed that, compared to the control group, the application of microbial agents reduced the relative abundance of *Fusarium* spp. by 66.18%. At the same time, the application of microbial agents also reduced the relative abundance of the fungus *Plectosphaerella* spp. by 79.90%, which is associated with delayed plant development. Additionally, the abundance of plant pathogens related to leaf blight and leaf spot, such as *Cercospora coniogrammes* and *Bipolaris drechsleri*, also increased. 

According to the FUNGuild functional predictions (Figure S5), the dominant fungi in the soil were pathotrophs and saprotrophs, with fewer symbiotrophs. Compared to the control group, microbial agent application reduced the proportion of plant pathogens and bryophyte parasites.

## 4. Discussion

China has consistently been the world’s largest peanut producer and ranks among the four major peanut-exporting countries globally. However, peanuts, as a cash crop, are susceptible to contamination by aflatoxins produced by *Aspergillus* spp., which poses significant risks to food safety and public health. This study aimed to develop a biocontrol agent for field application using atoxigenic *A. flavus* isolates screened from multiple provinces in China to achieve pre-harvest control.

The proportion of toxigenic and atoxigenic *A. flavus* varies globally. In this experiment, the proportion of atoxigenic *A. flavus* detected was 28%, which is close to the 27.69% found in Pakistan [39], higher than 13.4% in Brazil [40], but lower than 59% in Thailand [41] and 42% in India [42]. The selected atoxigenic *A. flavus* isolates not only exhibited an inhibition rate of up to 95% in laboratory competitive experiments but also effectively inhibited toxigenic *A. flavus* isolates under natural conditions. Prior to applying BCAs in peanut fields, *A. flavus* was screened from control soil in Tai’an and subjected to VCG analysis alongside four atoxigenic isolates. The results indicated that no hyphal fusion occurred between the field’s toxigenic *A. flavus* and the four atoxigenic isolates. Notably, hyphal fusion leading to genetic exchange is an exceedingly rare event in nature and is unlikely to be detected under field conditions. After the application of BCAs, the highest aflatoxin content in the soil measured 0.506 μg/kg, significantly below the international food and feed market limit of 4 μg/kg. Compared to the control group, aflatoxin levels in treated crops decreased by 18.78% to 69.35%, which was associated with the high occurrence of AF^−^ agent SSR characteristic primers. Following BCA application, the abundance of AF^−^ isolates in the soil increased, surpassing the proportion of toxigenic *A. flavus* aflatoxins, indicating that AF^−^ isolates in microbial preparations gained a competitive advantage in their ecological niche.

Among the five isolates of *A. flavus* selected in this study, CA04 and SZ05 were found not to produce sclerotia. Field experiments revealed that CA04, which does not produce sclerotia, may possess lower survival ability in outdoor environments compared to sclerotia-producing isolates like SF01 and GX61. This observation, based on the brief experimental period, suggested that non-sclerotium-producing AF^−^ isolates may have weaker stress resistance under field conditions. Further long-term studies are needed to assess the potential role of sclerotia production in enhancing the survival of AF^−^ isolates. This study found that under natural conditions, the applied atoxigenic isolates migrated and spread with environmental changes at a rate of 25 m/month. This suggested that BCAs may maintain biological control effects in areas beyond the application zone, providing preliminary insights into the dispersal rate of atoxigenic isolates in the soil following wind, rainfall, and insect activity, thereby laying the foundation for future scientific applications of microbial agents.

The application of atoxigenic *A. flavus* also reduced the relative abundance of *Fusarium* spp. and *Plectosphaerella* spp., known for root rot and late-season rot [43]. Peanut roots are less susceptible to rot, allowing rhizobia to efficiently attach to the roots and form nodules, facilitating nitrogen fixation and creating a favorable growth environment for the plant. Additionally, the use of microbial agents did not significantly affect the number of peanut nodules or the symbiotic nitrogen fixation between peanut plants and rhizobia in the soil, facilitating the conversion of atmospheric nitrogen into a usable form of nitrogen fertilizer for plants. However, it also presented potential risks: the fungus *Nothopassalora personata*, responsible for Late Leaf Spot (LLS), a common foliar fungal disease in peanuts, is known to cause yield reduction and defoliation [44]. Effective field control utilizing appropriate fungicides may help mitigate its spread. This experiment was conducted solely on the Huayu 39 peanut variety in Tai’an. To better evaluate the effects of biological control agents on peanut crops, future research should encompass a broader range of peanut varieties for comparative analysis.

## 5. Conclusions

The mixed biological control agent prepared from four atoxigenic *A. flavus* (CA04, SF01, GX61, and GX06) could effectively reduce aflatoxin pollution in peanut fields through competitive inhibition. VCG and SSR analysis revealed that the application of atoxigenic *A. flavus* does not result in hyphal fusion, and these atoxigenic *A. flavus* can survive in the soil for a long time, thus producing sustained biological control effects. Atoxigenic *A. flavus* can migrate in the field soil with changes in the environment, and this deepened our understanding of the population changes in the field after the application of atoxigenic *A. flavus*. OTU clustering biological classification of soil samples indicated that atoxigenic *A. flavus* may achieve comprehensive fungal management in peanut fields by reducing the species abundance of pathogenic fungi. This lays the foundation for the in-depth development and research of atoxigenic *A. flavus* biocontrol agents.

## Figures and Tables

**Table 1:** Screening time, region in China, and properties of partial *A. flavus* isolates in samples.

Year	Area	District	Sample	Isolate	VCG	AFB_1_ (μg/L)	AFB_2_ (μg/L)	CPA	Sclerotia
2022	Shandong	Qingdao	Corn	CA04	QC001	ND	ND	+	L
2022	Shandong	Qingdao	Corn	CA05	QC002	22.11 ± 0.09	575.23 ± 8.30	+	L
2022	Fujian	Zhangzhou	Soil	SZ01	ZS001	184.23 ± 12.65	6937.53 ± 31.67	+	L
2022	Fujian	Zhangzhou	Soil	SZ02	ZS001	94.37 ± 3.19	1049.32 ± 5.38	+	L
2022	Fujian	Zhangzhou	Soil	SZ03	ZS002	287.59 ± 2.67	969.24 ± 10.76	+	L
2022	Fujian	Zhangzhou	Soil	SZ04	ZS001	145.58 ± 3.87	1797.91 ± 32.89	+	L
2022	Fujian	Zhangzhou	Soil	SZ05	ZS003	ND	ND	+	L
2022	Fujian	Fuzhou	Soil	SF01	FS001	ND	ND	+	L
2022	Fujian	Fuzhou	Soil	SF02	FS002	162.8 ± 11.77	2564.1 ± 37.67	+	L
2022	Fujian	Fuzhou	Soil	SF03	FS003	1371.19 ± 40.23	20,767.48 ± 98.47	+	S
2023	Fujian	Fuzhou	Soil	SF04	FS004	168.45 ± 9.46	1069.79 ± 34.78	+	L
2022	Fujian	Qvanzhou	Soil	SQ01	QS001	168.4 ± 7.08	535.37 ± 18.75	+	L
2023	Fujian	Sanming	Soil	SM01	MS001	3231.64 ± 27.65	1592.78 ± 45.18	+	L
2023	Guangxi	-	Soil	GX06	GS001	ND	ND	−	L
2023	Guangxi	-	Soil	GX61	GS001	ND	ND	−	L
2023	Shandong	Tai’an	Soil	TA03	TS001	320.47 ± 5.62	6982.31 ± 65.39	+	L
2023	Shandong	Tai’an	Soil	TA04	TS001	468.54 ± 6.59	1889.73 ± 13.62	+	L
2023	Shandong	Tai’an	Soil	TA08	TS002	267.43 ± 3.55	4285.67 ± 54.87	+	L

ND: Not detected, +: CPA present, −: CPA absent, L: sclerotia diameter > 400 μm, S: sclerotia diameter < 400 μm.

## Data Availability

The raw reads were deposited into the NCBI Sequence Read Archive (SRA) database (Accession Number: PRJNA1057715).

## Supplementary Materials

The following supporting information can be downloaded at: https://www.mdpi.com/article/10.3390/jof11070491/s1, Figure S1: Plan of field test; Figure S2: Electrophoretic results of aflatoxin biosynthesis gene clusters; Figure S3: Thin-layer chromatographs visualized under 365 nm UV light for aflatoxin extracts from co-inoculation experiments; Figure S4: The sequencing to reflect the microbial diversity in soil samples; Figure S5: FunGuild fungal functional annotation of soil samples; Table S1: SSR amplification products of atoxigenic *A. flavus*; Table S2: Reduction of AFB_1_ content by co-inoculation with atoxigenic *A. flavus*.
