# Supplementary material for: Exploring the Characteristics of Atoxigenic Aspergillus flavus Isolates and Their Biocontrol Impact on Soil Fungal Communities
Source: J Fungi (Basel). 2025 Jun 27;11(7):491. doi: 10.3390/jof11070491 (PMC12300419; doi:10.3390/jof11070491)
Supplement: Supplementary file 1 [file jof-11-00491-s001.zip › jof-3669074-supplementary.pdf]

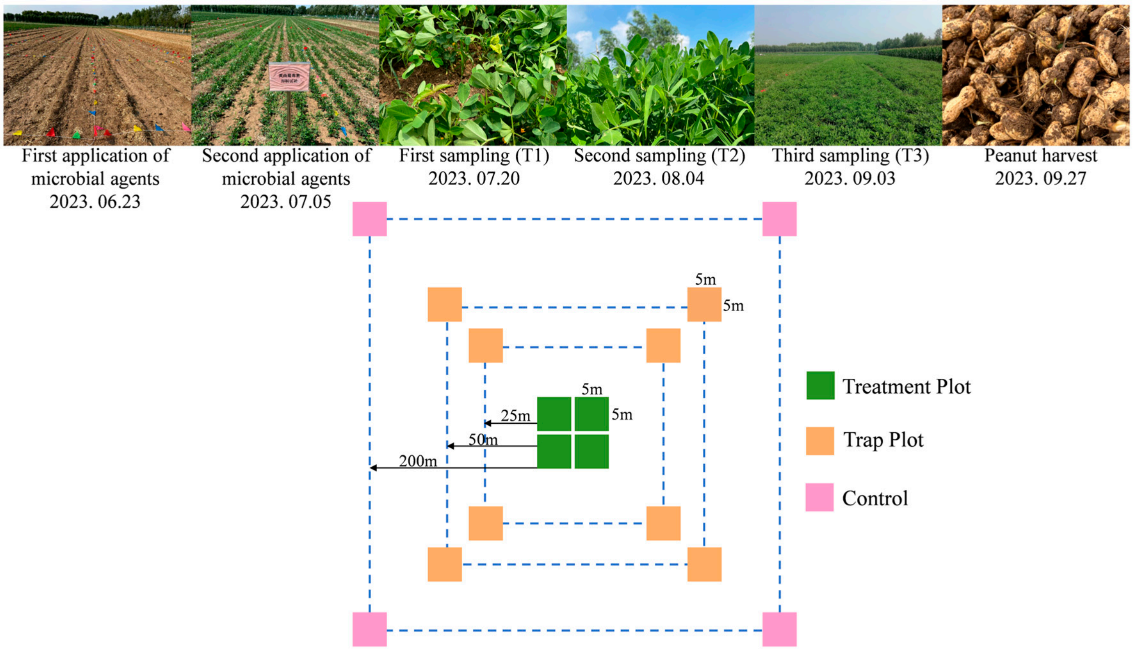

Figure S1. Plan of Field Test.

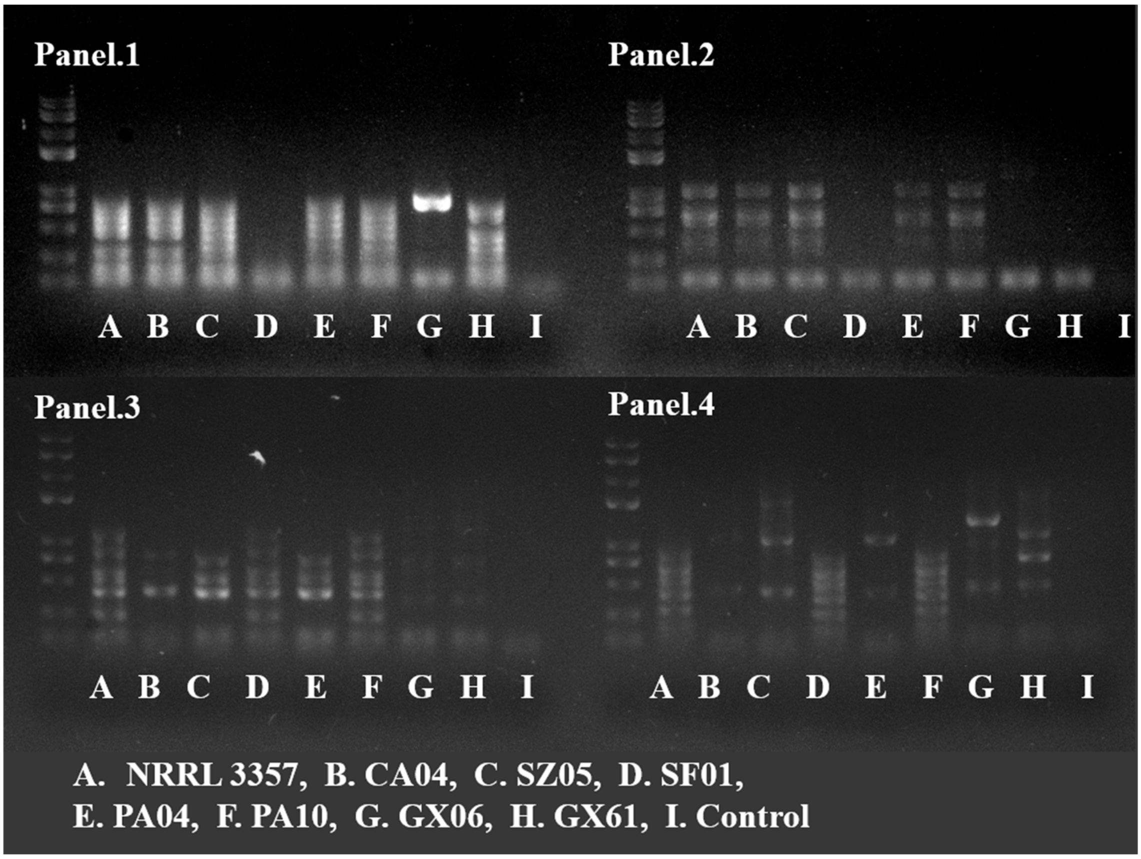

Figure S2. Electrophoretic results of gene clusters for aflatoxin biosynthesis gene clusters (the original image has been submitted).

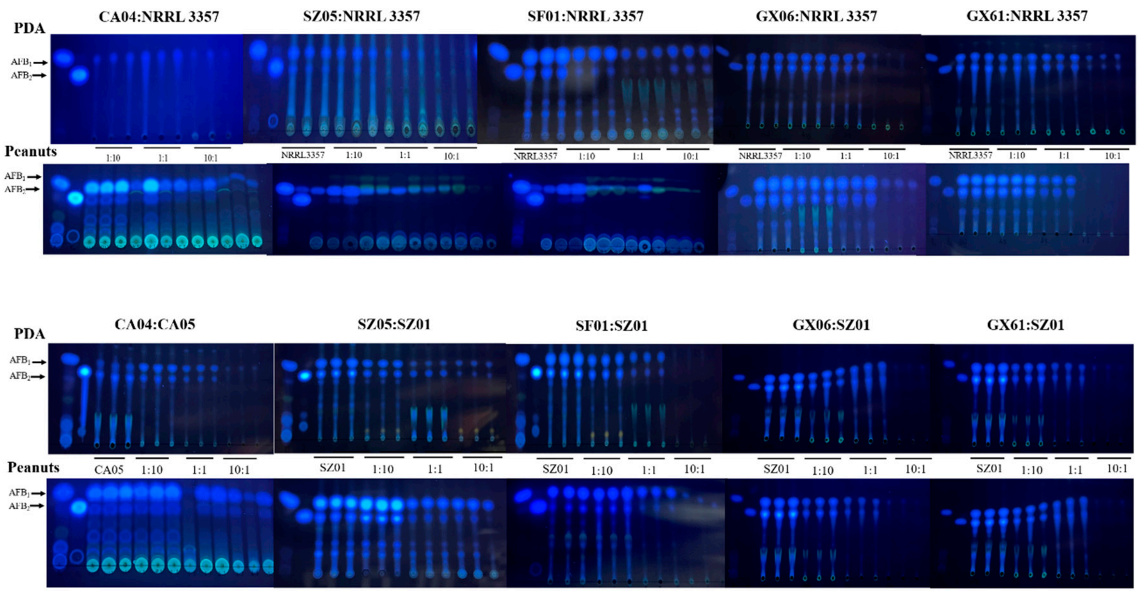

Figure S3. Thin layer chromatographs visualized under 365 nm UV light for aflatoxin extracts from co-inoculation experiments.

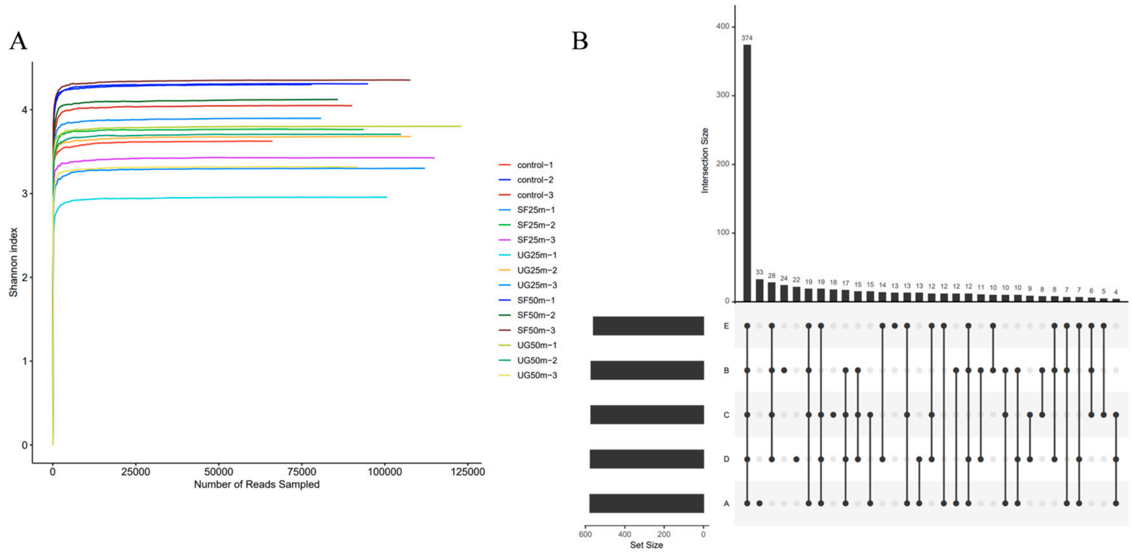

Figure S4. The sequencing to reflect the microbial diversity in soil samples.

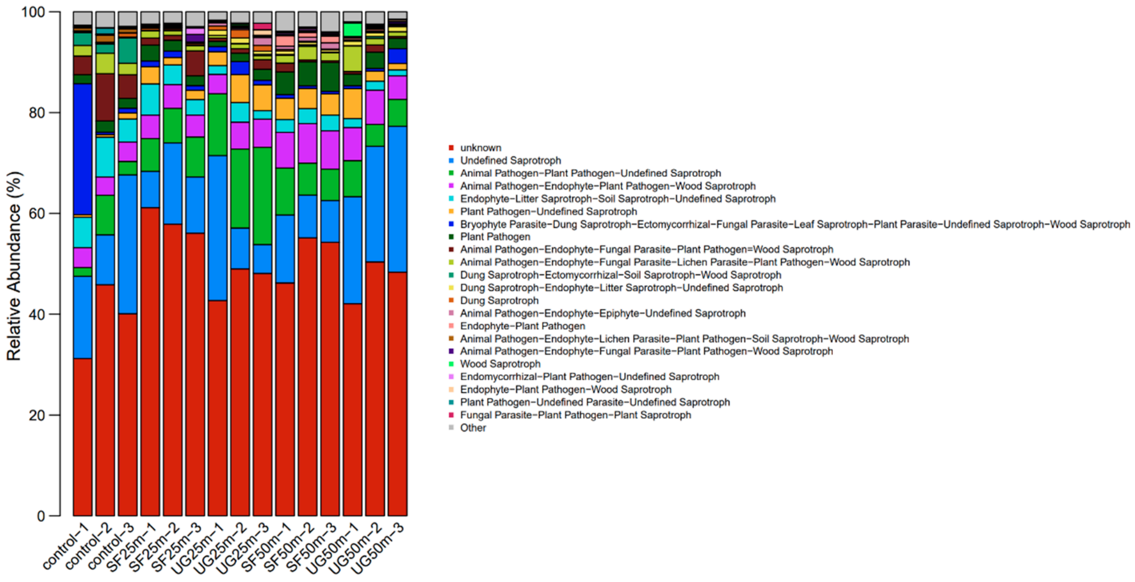

Figure S5. FunGuild fungal functional annotation of soil sample.

Table S1. SSR amplification products of atoxigenic *Aspergillus flavus*

Strains	PCR products size after Amplification, n=3																
	Panel A4					Panel B7				Panel D4				Panel E2			
	AF28	AF13	AF43	AF22	AF31	AF53	AF34	AF42	AF8	AF16	AF54	AF17	AF11	AF66	AF64	AF63	AF55
CA04	135/135	138/162	382/382	339/367[a]	338/338	139/154	304/370	241/241[b]	0/0	179/185	179/188	373/373	143/160	273/273	204/217	133/133	192/199
CA05	118/132	130/151	397/431	179/179	332/332	147/165	306/306	181/200	0/0	190/190	167/175	360/360	203/225	273/282	206/219	134/134	182/188
SZ01	132/132	140/157	387/417	179/179	329/329	147/165	305/305	167/215	0/0	179/190	180/188	359/359	142/160	273/273	206/219	134/134	178/193
SZ02	131/131	172/183	391/424	179/179	328/328	143/143	306/362	0/0	0/0	0/0	167/176	359/359	145/145	0/0	173/173	134/134	174/204
SZ03	145/158	130/140	398/398	142/142	369/369	146/146	0/0	168/168	307/385	177/182	180/180	394/394	187/187	274/274	180/202	138/138	192/199
SZ04	148/161	163/180	391/421	183/183	360/360	137/137	334/351	158/205	0/0	180/185	167/174	360/468	140/140	283/283	239/246	134/134	185/190
SZ05	130/130	141/158	394/394	179/179	354/385	141/160	292/315	196/214	519/519	178/185	166/176	371/385	195/215[a]	270/270	183/183	137/148	174/174[b]
SF01	130/130	141/158	394/424[b]	179/179	354/385	141/156	292/317	196/214	519/519	178/178	178/178	372/372	179/197[a]	270/270	183/183	137/137	174/190
SQ01	142/142	144/161	390/419	179/179	325/325	141/156	313/341	188/205	519/519	178/189	159/159	369/384	145/151	273/279	184/184	133/133	181/181
SM01	141/141	138/152	398/430	179/179	393/437	150/167	307/328	222/222	519/519	270/270	178/178	371/386	142/160	273/273	203/213	133/133	174/174
SF02	118/118	136/144	404/439	142/142	325/348	141/156	306/324	162/162	520/520	169/169	165/175	410/425	145/163	229/280	203/214	132/132	163/163
SF04	139/139	147/165	382/382	187/187	310/310	150/150	317/339	176/191	522/522	178/185	170/170	360/374	145/163	0/0	172/172	137/137	0/0
GX06	122/122	130/130	413/413	39/162[b]	314/314	136/149	306/306	144/144[a]	0/0	169/169	165/165	378/378	0/0	0/0	167/167	132/132	182/182
GX61	140/140	155/155	404/483	38/189	350/350	155/155	323/338	175/175[b]	0/0	179/185	165/165	385/393	0/0	284/284	190/193	133/133	213/213[a]

a. primary amplification primers used during recovery, b. auxiliary amplification primers used during recovery.

Table S2. Reduction of AFB_1 content by co-inoculation with atoxigenic *A. flavus*

Strains	PDA（10 d）		Peanuts（14 d）	
	AFB_1(μg/L)	Inhibition rate(%)	AFB_1(μg/L)	Inhibition rate(%)
CA04:NRRL 3357[b]	5,143.2±154.3	39.9	5,274.2±265.7	35.1
CA04:NRRL 3357[c]	5,352.6±224.1	37.4	2,673.3±210.1	67.1
CA04:NRRL 3357[d]	1,681.4±88.2	80.3	2,036.2±88.2	75.0
SF01:NRRL 3357[b]	5,930.8±837.5	27.8	2,838±349.2	66.9
SF01:NRRL 3357[c]	3,920.0±118.0	52.3	2,326.7±130.5	72.9
SF01:NRRL 3357[d]	2,884.7±587.7	64.9	1,048.9±131.7	87.8
SZ05:NRRL 3357[b]	5,702.4±116.6	33.3	5,643.3±756.5	30.6
SZ05:NRRL 3357[c]	4,259.9±359.0	50.2	3,200.0±921.5	60.6
SZ05:NRRL 3357[d]	1,531.3±104.5	82.1	2,870.0±292.0	64.7
GX06:NRRL 3357[b]	7,426.2±608.7	29.3	7,817.3±597.9	30.1
GX06:NRRL 3357[c]	4,518.7±393.2	57.1	5,360.4±364.7	52.0
GX06:NRRL 3357[d]	0.0	100.0	856.2±278.6	92.7
GX61:NRRL 3357[b]	8,792.4±301.3	16.5	10,236.9±278.6	8.0
GX61:NRRL 3357[c]	6,970.8±524.3	34.5	4,429.8±646.9	60.9
GX61:NRRL 3357[d]	1,716.4±386.9	84.2	1,824.0±411.2	84.6
CA04:CA05[b]	429.5±19.8	25.3	6,112.3±228.5	11.9
CA04:CA05[c]	345.4±8.0	40.6	3,052.6±308.8	56.0
CA04:CA05[d]	188.6±6.2	67.2	54.3±63.8	98.4
SF01:SZ01[b]	3,201.9±21.9	53.8	6,166.8±229.3	11.1
SF01:SZ01[c]	1,346.0±35.7	80.6	1,886.5±83.5	72.8
SF01:SZ01[d]	502.2±130.3	92.8	114.5±64.4	98.3
SZ05:SZ01[b]	6,395.6±168.8	7.8	6,754.6±423.5	2.6
SZ05:SZ01[c]	6,263.0±87.5	9.7	4,666.9±601.5	32.7
SZ05:SZ01[d]	5,367.4±219.1	22.6	1,670.9±362.6	75.9
GX06:SZ01[b]	12,225.2±357.2	-16.5	13,587.5±931.4	-22.8
GX06:SZ01[c]	4,939.1±477.7	53.6	5,360.4±364.7	52.1
GX06:SZ01[d]	1,891.6±535.8	82.3	2,605.8±786.1	77.4
GX61:SZ01[b]	9,598.0±1001.8	9.5	9,678.5±885.6	13.4
GX61:SZ01[c]	3,923.3±729.8	63.7	4,243.7±794.9	62.4
GX61:SZ01[d]	2,031.7±602.7	81.6	1,898.5±328.8	83.1
